# *Portulaca Oleracea* L. (Purslane) Extract Protects Endothelial Function by Reducing Endoplasmic Reticulum Stress and Oxidative Stress through AMPK Activation in Diabetic Obese Mice

**DOI:** 10.3390/antiox12122132

**Published:** 2023-12-18

**Authors:** Lingchao Miao, Chunxiu Zhou, Haolin Zhang, Meng Sam Cheong, Yi Tan, Yuehan Wang, Xutao Zhang, Hua Yu, Wai San Cheang

**Affiliations:** Institute of Chinese Medical Sciences, State Key Laboratory of Quality Research in Chinese Medicine, University of Macau, Avenida da Universidade, Taipa, Macau 999078, China

**Keywords:** purslane, diabetes mellitus, endoplasmic reticulum stress, endothelial function, nitric oxide, reactive oxygen species

## Abstract

*Portulaca oleracea* L. (purslane) is a food and a traditional drug worldwide. It exhibits anti-inflammatory, anti-oxidative, anti-tumor, and anti-diabetic bioactivities; but its activity on diabetic-associated endothelial dysfunction is unknown. This study aimed to investigate the effect of purslane on endothelial function and the underlying mechanisms. Male C57BL/6 mice had 14-week ad libitum access to a high-fat rodent diet containing 60% kcal% fat to induce obesity and diabetes whereas purslane extract (200 mg/kg/day) was administered during the last 4 weeks via intragastric gavage. Primary rat aortic endothelial cells and isolated mouse aortas were cultured with a risk factor, high glucose or tunicamycin, together with purslane extract. By ESI-QTOF-MS/MS, flavonoids and their glycoside products were identified in the purslane extract. Exposure to high glucose or tunicamycin impaired acetylcholine-induced endothelium-dependent relaxations in aortas and induced endoplasmic reticulum (ER) stress and oxidative stress with the downregulation of 5′ AMP-activated protein kinase (AMPK)/ endothelial nitric oxide synthase (eNOS) signaling. Co-incubation with purslane significantly ameliorated these impairments. The effects of purslane were abolished by Compound C (AMPK inhibitor). Four-week purslane treatment ameliorated aortic relaxations, ER stress, and oxidative stress in diabetic obese mice. This study supported that purslane protected endothelial function, and inhibited ER stress and oxidative stress in vasculature through AMPK/eNOS activation, revealing its therapeutic potential against vascular complications in diabetes.

## 1. Introduction

Type 2 diabetes mellitus, an inflammatory metabolic disease is characterized by several metabolic abnormalities, including hyperglycemia, insulin resistance, excess secretion of free fatty acids, and hyperinsulinemia [1]. Various complications of diabetes, especially vascular complications, lead to mortality in diabetic patients. Many factors modulate diabetes distress and the health literacy of diabetic patients [2,3]. Cardiovascular homeostasis is modulated by vascular endothelial cells, which secrete both vasodilators and vasoconstrictors including endothelium-derived relaxing factors (EDRFs), endothelium-derived contracting factors (EDCFs), prostaglandins and endothelium-derived hyperpolarizing factors (EDHFs). Nitric oxide (NO) is one of the most powerful EDRFs. In endothelial cells, the synthesis of NO is catalyzed by endothelial nitric oxide synthase (eNOS), which is critical for the maintenance of vascular function [4]. Nevertheless, it is generally agreed that hyperglycemia results in decreased NO bioavailability, leading to impaired endothelium-dependent vasodilation [5]. Furthermore, 5′ AMP-activated protein kinase (AMPK) plays an important role in maintaining energy homeostasis and modulating various physiological processes in the cardiovascular system [6]. AMPK has multiple downstream targets like eNOS. Emerging evidence has revealed the importance of AMPK activity in modulating oxidative stress and NO bioavailability, affecting vascular function [7].

The endoplasmic reticulum (ER) is a cellular organelle involved in the folding, maturation, and trafficking of proteins and reactions early to cellular stresses [8]. ER stress can be caused by various risks, including hyperglycemia, oxidative stress, hypoxia, energy deprivation, and stimulation by chemicals like tunicamycin and thapsigargin [9]. Normally, the proper folding of transmembrane and secretory proteins relies on the ER intraluminal oxidative environment and high calcium concentration; nevertheless, a disturbed ER environment will result in the accumulation of unfolded proteins in the ER lumen, which triggers the activation of unfolded protein response (UPR). Three transmembrane proteins consisting of protein kinase RNA-like ER kinase (PERK), inositol-requiring enzyme 1 (IRE1), and activating transcription factor 6 (ATF6) with the corresponding downstream signaling cascade are stimulated. UPR is initially an adaptive pathway to repair the homeostasis in ER. Prolonged accumulation of unfolded proteins leads to ER stress, which mediates the development and progression of endothelial dysfunction, for example, by lowering NO bioavailability, increasing the generation of reactive oxygen species (ROS), and inducing inflammation in vasculature [10,11].

*Portulaca oleracea* L. is both a food and medicinal herb. More commonly, it is also called purslane in USA or Australia, Ma-Chi-Xian in China, pigweed in England, and pourpier in France [12]. In present times, purslane has been proven to exhibit abundant pharmacological benefits, such as hepatoprotective [13], anti-obesity and anti-diabetic [14,15,16,17], and anti-inflammatory and anti-oxidant effects [18,19]. Along with its effects that improve glucose and lipid metabolism, a previous study has revealed the vascular protective effect of purslane to ameliorate endothelial dysfunction as well as vascular inflammation in diabetic db/db mice [20]. However, the underlying mechanism remains to be explored. Here in this study, we aimed to investigate whether purslane extract treatment protected against diabetes-associated endothelial-dysfunction both in vitro and in vivo and determine the possible mechanisms.

## 2. Materials and Methods

### 2.1. Materials

RPMI1640 and low-glucose DMEM medium powder, FBS supplement, penicillin-streptomycin solution (10,000 U/mL), and trypsin-EDTA solution (0.5%) were purchased from Gibco (Carlsbad, CA, USA). Bovine serum album (BSA), thiazolyl blue tetrazolium bromide (MTT), collagenase type 1A, Compound C (Cpd C, AMPK inhibitor), phenylephrine (Phe), acetylcholine (ACh), N^G^-nitro-L-arginine methyl ester (L-NAME, NOS inhibitor), and sodium nitroprusside (SNP, NO donor) were purchased from Sigma Aldrich (St. Louis, MO, USA). Fluorescence dye, dihydroethidium (DHE) was obtained from Invitrogen (Carlsbad, CA, USA). 

### 2.2. Preparation and Identification of the Purslane Extract

Whole plants were collected from Anhui, China. First, 10 g of shade-dried plants was chopped into small pieces, which were transferred to an electric grinder to be smashed for 5 min into a fine powder; the powder was then immersed in 100 mL water-ethanol (1:1, *v*/*v)* overnight. The solution was then collected and another 100 mL water-ethanol (1:1, *v*/*v*) was added to the powder along with an ultrasonic bath. Afterward, the mixed extracts were filtered and concentrated under rotate reduced pressure to remove ethanol. After being frozen at −80°C overnight, the concentrated extracts were lyophilized by a Virtis Freeze Dryer (Te Virtis Company, Los Angeles, CA, USA), and the final freeze-dried powder of purslane extract was obtained. The powder was dissolved in PBS and the extract solution was filtered by 0.22 μm sterile filters before its application to cultured aortas or cells.

The purslane extract was identified using UPLC-ESI-QTOF-MS/MS. Liquid chromatographic separation was performed on a Waters ACQUITY UPLC H-Class system (Waters, Milford, MA, USA) equipped with a Waters ACQUITY UPLC BEH C18 column (2.1 mm × 50 mm, 1.7 µm). The column temperature was set as 30 °C. The mobile phase consisted of 0.1% formic acid in water (phase A) and acetonitrile (phase B). The flow rate was set as 0.3 mL/min. The injection volume was 1 µL. Samples were eluted with a linear gradient: 0–1 min, 5% B; 1–28 min, 5–95% B; 28–36 min, 95–5%; 36–40 min, 5% B.

The MS analysis was studied on a Waters XEVO G2-XS QTOF system combined with an electrospray ionization (ESI) source (Waters, MA, USA). The MSE Centroid mode under sensitivity and negative electrospray ionization (ESI-) mode were used to collect data within a range of 10 to 1500 Da. The scan time was set at 1 s. Tuning parameters included capillary voltage of 2 kV, sampling cone voltage of 40 V, source Offset of 80 V, source temperature of 120 °C, desolvation temperature of 450 °C, cone gas flow of 50 L/h, and desolvation gas flow of 700 L/h. Data were processed by MassLynx V 4.1 software (Waters, Milford, MA, USA).

### 2.3. Animal Experiments

The use of mice and rats for this study was consistent with the ethics application approved by the Animal Research Ethical Committee at the University of Macau. All animal experiments complied with the ARRIVE guidelines and were carried out in accordance with the National Research Council’s Guide for the Care and Use of Laboratory Animals. Male C57BL/6J mice and Sprague Dawley (SD) rats were held at constant temperature (22–23 °C) at an alternating 12 h light/dark cycle. Six-week-old mice had ad libitum access to a high-fat diet (60% kcal% fat) for 10 weeks followed by oral administration of vehicle (0.3% sodium carboxymethyl cellulose CMC-Na solution) and purslane extract at 400 mg/kg/day for another 4 weeks. Mice given ad libitum access to a normal chow diet and water served as control. SD rats were all fed with a normal chow diet and sacrificed for primary culture of aortic endothelial cells for in vitro experiments.

During the last few days of the experiment, systolic blood pressure (SBP) and diastolic blood pressure (DBP) of mice from different groups were measured by a tail-cuff method with the use of CODA noninvasive blood pressure system (Kent Scientific Corporation, Torrington, CT, USA).

After 6 h fasting, the mouse tail was cut by surgical scissors to collect blood droplets for the measurement of fasting blood glucose (FBG) level with a commercial glucometer. To perform an oral glucose tolerance test (OGTT), mice were weighed and orally administered glucose solution (1.2 g/kg body weight) after 6 h fasting. Blood glucose levels at time intervals of 0, 15, 30, 60, 90, and 120 min were then measured. To perform the insulin tolerance test (ITT), mice were also weighed and injected intraperitoneally with insulin (0.5 U/kg body weight) after 2 h fasting. Blood glucose levels of mice were determined by a glucometer at the same six time intervals aforementioned.

### 2.4. Functional Assay by Wire Myograph

Mouse aortic segments about 2 mm long were accessed for isometic force measurement by Multi Myograph System (Danish Myo Technology, Hinnerup, Denmark) as previously described [21]. The aortic rings were suspended in the myograph chamber containing 5 mL Krebs solution by fixing the two steel wires through the vessul lumen to the jaws. The organ chambers were gassed by 95% O_2_ plus 5% CO_2_. An optimal baseline tension of the rings was adjusted to 3 mN, and the rings were then stabilized for 60 min. Then, 60 mM of KCl solution was added to induce contraction in arteries and was then washed away. In the first set of experiments, the rings were contracted by phenylephrine (Phe, 3 μM) and cumulatively relaxed by acetylcholine (ACh, increasing concentrations from 3 nM to 10 μM). The alterations of endothelium-dependent relaxations (EDRs) were thus recorded. The second set was to examine endothelium-independent relaxations. To remove the interference of endothelium-derived NO, 100 μM N^G^-nitro-L-arginine methyl ester (L-NAME, a non-selective NOS inhibitor) was used to treat the rings for 30 min. After contraction induced by Phe, relaxations of the aortic rings triggered by NO donor, sodium nitroprusside (SNP, cumulatively from 1 nM to 10 μM) were recorded.

### 2.5. Ex vivo Culture of Mouse Aortas

Mouse thoracic aortas were immediately isolated after the mice were sacrificed and were then cleaned from surrounding fat or adjacent tissue in sterile phosphate-buffered saline (PBS). The isolated aortas were cultured in DMEM with 10% FBS and 1% penicillin-streptomycin solution at 37 °C. High glucose (HG, 30 mM), tunicamycin (Tuni, 2 μg/mL), purslane extract (100 or 400 μg/mL), and Compound C (Cpd C, 5 μM) were added into the culture medium individually. The aortic segments of high glucose groups were incubated at 37 °C for 48 h whereas those of tunicamycin groups were incubated for 24 h. After treatment, the aortic segments were collected for further studies including functional test with a wire myograph, mechanism exploration with Western blotting assay, and immunofluorescence staining assay. The culture medium was also collected for the measurement of NO release by the segments.

### 2.6. Primary Culture of Rat Aortic Endothelial Cells (RAECs)

The rat thoracic artery was rapidly isolated from adjacent tissue and cut longitudinally in sterile PBS after the rat was euthanized by CO_2_ suffocation. The aorta stripe was incubated and digested by sterile collagenase type 1A dissolved in PBS (2 mg/mL) for 15 min at 37 °C along with a gentle shake. The detached RAECs were obtained via centrifugation (2500 rpm, 10 min) and resuspended in RPMI-1640 medium containing 10% FBS and 1% penicillin–streptomycin solution. The medium was refreshed after 1 h incubation so that the unattached cells were removed. RAECs were cultured till 80–90% confluency in a humidified incubator with 5% CO_2_ at 37 °C.

### 2.7. Western Blotting

Whole lysate isolated from tissue samples was obtained by using ice-cold RIPA lysis buffer (Beyotime, Shanghai, China) supplemented with a complete protease inhibitor cocktail (Roche, St. Louis, MO, USA) and PhosSTOP phosphatase inhibitors (Roche, St Louis, MO, USA) after the aortas were snap frozen by liquid nitrogen. The lysate from cellular samples was obtained by using ice-cold RIPA lysis buffer containing 1% Phosphatase Inhibitor Cocktail (Thermo, Carlsbad, CA, USA) and 1% phenylmethylsulfonyl fluoride (PMSF; Thermo, Carlsbad, CA, USA). The homogenates were incubated for 30 min on ice and were then centrifuged at 15,000 rpm for 20 min at 4 °C. The supernatants were collected, and protein concentrations were determined by a BCA protein assay kit (Beyotime, Shanghai, China). An equal amount of proteins was loaded and separated by SDS-PAGE gels (8% or 10%) and transferred to PVDF membranes (Millipore, Billerica, MA, USA). Being blocked by 1% BSA in TBST buffer at ambient temperature for 2 h, membranes were incubated with the primary antibodies against target proteins overnight at 4 °C. Membranes were washed by TBST three times and then incubated with corresponding HRP-conjugated secondary antibodies for 2 h at room temperature. Bands on membranes were detected by a Supersignal^TM^ West Femto Maximum Sensitivity Substrate (Thermo Scientific, Waltham, MA, USA) in a ChemiDoc^TM^ MP Imaging System (BIO-RAD, Hercules, CA, USA). Various primary antibodies used in this study consisted of phospho-eNOS (Ser1177) rabbit mAb (1:500), eNOS rabbit mAb (1:1000), phospho-AMPKα (Thr172) rabbit mAb (1:1000), AMPKα rabbit mAb (1:500), phospho-eIF2α (Ser51) rabbit mAb (1:1000), eIF2α rabbit mAb (1:500), ATF6 rabbit mAb (1:1000), XBP1 rabbit mAb (1:1000), CHOP (L63F7) mouse mAb (1:1000), and GAPDH rabbit mAb (1:3000). All of these primary antibodies were purchased from Cell Signaling Technology (Danvers, MA, USA), except for anti-ATF6 and anti-XBP1 antibodies from Abcam (Cambridge, UK).

### 2.8. Measurement of NO Release

NO release levels in the medium collected from cultured RAECs were detected by the Griess reagent kit (Invitrogen, Carlsbad, CA, USA) following the manufacturer’s instructions. Absorbance was read by a SpectraMax M5 microplate reader (Molecular Devices, Silicon Valley, CA, USA) at 548 nm.

### 2.9. ROS Determination by Dihydroethidium (DHE) Staining

Mouse aortic segments embedded in the OCT compound with cut into (10 μm thick sections) using a Leica CM 1000 cryostat. Slides with aortic sections and cultured RAECs in 24-well plates were incubated for 30 min in 5 μM DHE-containing normal physiological saline solution (NPSS) in the dark at 37 °C. NPSS contained the following compounds: 140 mM NaCl, 5 mM KCl, 1 mM MgCl_2_, 1 mM CaCl_2_, 10 mM glucose, and 5 mM HEPES (pH 7.4). DHE fluorescence images were captured by Leica-DMi8 inverted fluorescent microscope and the fluorescence intensity was analyzed.

### 2.10. Statistical Analysis

Results from n separate experiments were all analyzed and shown as mean values with standard error of the mean (SEM). Running GraphPad Prism software version 8.0 (GraphPad Software, San Diego, CA, USA), data from different groups were analyzed by one-way analysis of variance (ANOVA) and unpaired Student *t*-test. *p* < 0.05 indicates a statistically significant difference.

## 3. Results

### 3.1. Identification Results of Purslane Extract

**Figure 1:**
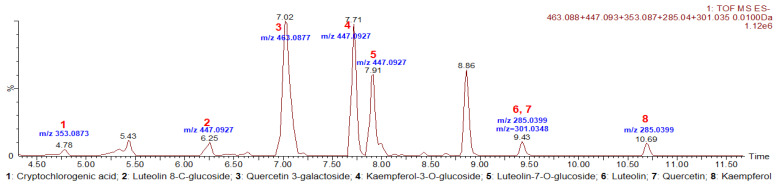
Extracted ion chromatogram (EIC) of identified components in the purslane extract. EIC of polyphenols for 1: Cryptochlorogenic acid;2: Luteolin 8-C-glucoside; 3: Quercetin 3-galactoside; 4: Kaempferol-3-O-glucoside; 5: Luteolin-7-O-glucoside; 6: Luteolin; 7: Quercetin; and 8: Kaempferol.

There are eight kinds of polyphenols identified in the purslane extract, shown as extracted ion chromatograms (EIC) in Figure 1. The information about the identification of eight constituents in the purslane extract by ESI-QTOF-MS/MS (ESI-) was listed in Table 1, including retention time, observed mass, theoretical mass, predicted chemical formula, and observed MS/MS fragment ions. The identification results and basis are also listed in Table 1. Cryptochlorogenic acid, luteolin 8-C-glucoside, quercetin 3-galactoside, luteolin, quercetin, and kaempferol were identified, respectively, by matching with standard substances. Kaempferol-3-O-glucoside and luteolin-7-O-glucoside were matched with the MS/MS results in the PubChem database. 

### 3.2. Purslane Extract Treatment Ameliorates Endothelial Dysfunction and Glucose Metabolism Disorder in DIO Mice

**Figure 2:**
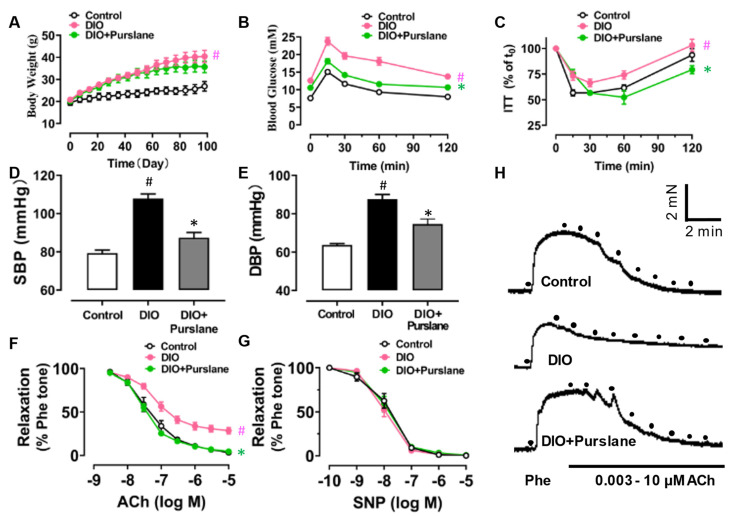
Purslane extract improves endothelial dysfunction and diabetes symptoms in diet-induced obese (DIO) mice. (**A**) Body weight changes of C57 BL/6J mice with standard chow or high-fat diet for a total of 14 weeks and 4-week oral treatment with vehicle (0.3% CMC-Na solution) or purslane extract (200 mg/kg/day). (**B**) Oral glucose tolerance test (OGTT) results after fasting for 6 h. (**C**) Insulin tolerance test (ITT) results after fasting for 2 h. (**D**,**E**) Systolic (SBP) and diastolic (DBP) blood pressure changes in all groups of mice detected by the tail-cuff method. (**F**) Summarized data revealing the effect of 4-week purslane treatment on endothelium-dependent relaxations (EDRs) induced by acetylcholine (ACh), and (**G**) sodium nitroprusside(SNP)-induced endothelium-independent relaxations in all groups of mouse aortas. (**H**) Representative traces of ACh-induced EDRs in mouse aortas in vivo. Data are shown as mean ± SEM (*n* = 5). # *p* < 0.05 DIO vs. Control; ∗ *p* < 0.05 DIO + Purslanevs. DIO.

In order to find out the effect of purslane extract on vascular function in vivo, a 4-week oral administration of purslane extract (200 mg/kg/day) was performed on diet-induced obese (DIO) mice. The body weight of DIO mice was obviously increased due to the high-fat diet feeding for a total of 14 weeks, compared with the control mice, which were fed with standard chow. However, purslane extract treatment only showed a minor but not significant effect on the body weight increase of DIO mice (Figure 2A). Fasting glucose levels of DIO mice were over 11.1 mM (value at t_0_ of oral glucose tolerance test), much higher than those of control mice, standing for the successful establishment of a diabetic model in mice (Figure 2B). Not only this, but the oral glucose tolerance test (Figure 2B) also reveals that the glucose sensitivity of DIO was impaired compared to lean control mice. Moreover, insulin irresponsiveness was discovered in DIO mice using an insulin tolerance test (Figure 2C). After a 4-week purslane extract treatment, both insulin sensitivity and glucose tolerance were significantly improved in DIO mice. The elevated systolic and diastolic blood pressures of DIO mice were markedly normalized by 4-week purslane extract treatment (Figure 2D–E). Without effects on SNP-induced endothelium-independent relaxations in aortas, 4-week purslane extract administration significantly restored the ACh-induced EDRs in DIO mice (Figure 2F–H).

In addition, hyperlipidemia was proved in DIO mice where the plasma levels of total cholesterol, triglyceride, and low-density lipoprotein (LDL) were elevated, compared with the lean control mice (Table 2). Four-week oral administration of purslane extract helped to decrease the total cholesterol, triglyceride, and LDL concentrations in plasma, even though the positive effect on plasma high-density lipoprotein (HDL) levels was insignificant.

### 3.3. Purslane Improves Endothelial Function via Activation on AMPK/eNOS Signaling and Suppression on ER Stress

**Figure 3:**
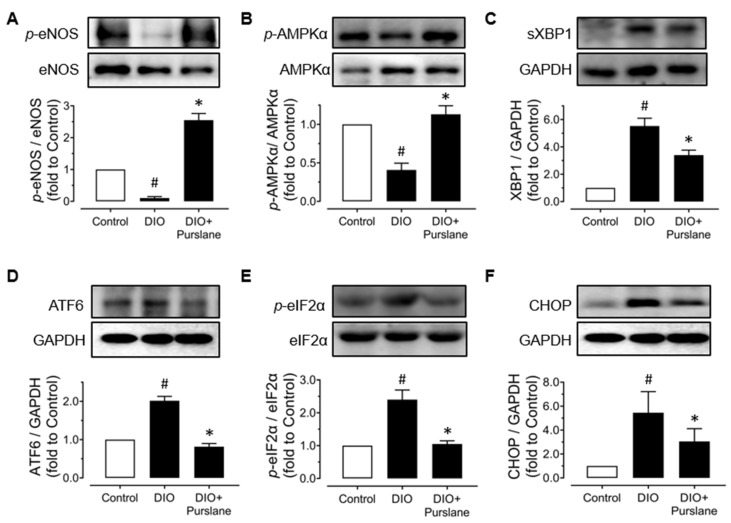
Purslane extract activates AMPK/eNOS signaling pathway and suppresses endoplasmic reticulum (ER) stress in DIO mice. Representative Western blotting images and summarized graphs showing (**A**) eNOS phosphorylation at Ser1177 and total eNOS (140 kDa), (**B**) phosphorylation of AMPKα at Thr172 and total AMPKα (62 kDa) expression levels, and ER stress markers including (**C**) spliced XBP1 (56 kDa), (**D**) cleaved ATF6 (50 kDa), (**E**) phosphorylation of eIF2α at Ser52 and total eIF2α expression levels (38 kDa) and (**F**) CHOP (27 kDa) compared to GAPDH in aortas of lean control, DIO and purslane extract (200 mg/kg/day, 4 weeks) treated DIO mice. Data are shown as mean ± SEM (*n*= 4–5). # *p*< 0.05 DIO vs. Control; ∗ *p*< 0.05 DIO + Purslane vs. DIO.

In order to further investigate the potential mechanisms of the endothelial protective effect of purslane extract in vivo, aortas were isolated from all groups of mice to examine protein expressions using Western blot. The expression levels of phosphorylated eNOS (at Ser1177) and phosphorylated AMPKα (at Thr172) were all decreased apparently in DIO mice compared with lean mice, whilst 4-week treatment of purslane extract effectively enhanced these expressions (Figure 3A,B). Moreover, ER stress was significantly inhibited by purslane treatment as the DIO-induced expressions of representative ER stress markers including spliced X-box binding protein 1 (sXBP1), cleaved ATF6, phosphorylated eukaryotic initiation factor-2α (eIF2α) at Ser52 and CHOP were reduced by purslane extract in aortas (Figure 3C–F).

### 3.4. Purslane Extract Activates AMPK/eNOS Pathway and Alleviates ER Stress in High Glucose/Tunicamycin-Treated Mouse Aortas to Protect Endothelial Function

**Figure 4:**
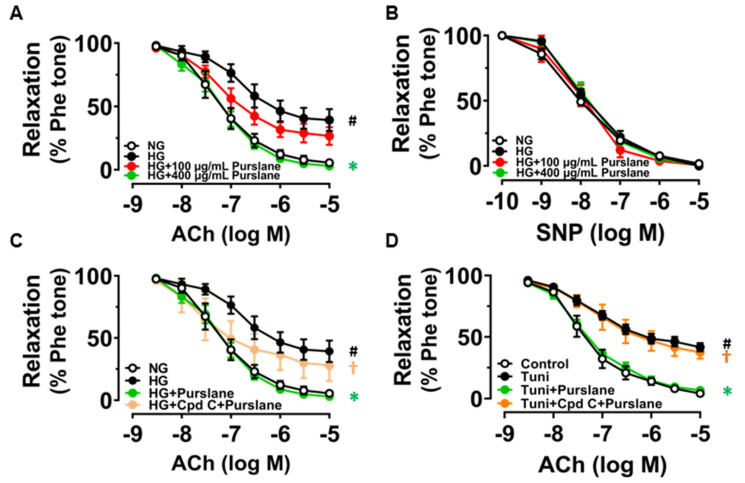
Purslane extract protects against high glucose- and tunicamycin-triggered endothelial dysfunction on AMPK activation. (**A**) Summarized data of the effects of purslane extract (100 and 400 μg/mL) on ACh-induced EDRs and (**B**) SNP-induced relaxations in mouse aortas exposed to high glucose (HG, 30 mM, 48 h). (**C**,**D**) Effect of AMPK inhibitor Compound C (Cpd C, 5 μM) on ACh-induced EDRs in mouse aortas co-treated with high dose purslane extract (400 μg/mL) under the high glucose (HG, 30 mM, 48 h) or tunicamycin (Tuni, 2 μg/mL, 24 h) stimulated condition. Data are shown as mean ± SEM (*n* = 4). # *p* < 0.05 HG vs. NG (5.55 mM glucose with mannitol added as osmotic control) or Tuni vs. Control; ∗ *p* < 0.05 vs. HG or Tuni; † *p* < 0.05 vs. HG+ Purslane or Tuni+ Purslane.

Aortic rings isolated from C57BL/6J mice were treated with high glucose (30 mM, 48 h) to mimic the hyperglycemic condition in diabetes. The ACh-induced endothelium-dependent relaxations (EDRs) were remarkably impaired by high glucose stimulation while treatment with purslane extract at 400 μg/mL reversed the impairment (Figure 4A). However, purslane extract at a lower dose of 100 μg/mL did not exert significant improvement against HG-induced endothelial dysfunction. SNP-induced endothelium-independent relaxations were all unchanged among groups, which meant the vascular smooth muscle could normally respond to NO (Figure 4B). Moreover, such protective effect was abolished by AMPK inhibitor Compound C (Cpd C, 5 μM) (Figure 4C). Likewise, the mouse aortas were stimulated with tunicamycin (Tuni, 2 μg/mL) with or without the co-treatment of purslane extract (400 μg/mL) for 24 h. Impaired EDRs caused by tunicamycin were restored by the treatment of purslane extract and the protective effect was inhibited by Compound C (Figure 4D).

**Figure 5:**
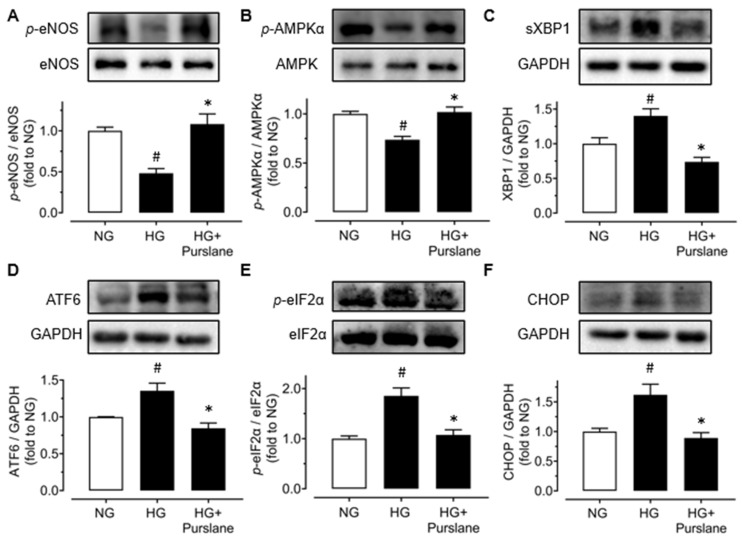
Purslane extract activates AMPK/eNOS signaling and inhibits ER stress in high glucose-induced mouse aortas (mimic hyperglycemia). Representative Western blotting images and analyzed graphical results for (**A**) phospho (p)-eNOS at Ser1177 (140 kDa) and (**B**) phospho (p)-AMPKα at Thr172 (62 kDa) in ratio to their respective total proteins in aortic segments stimulated with high glucose (HG, 30 mM, 48 h) with or without the co-treatment of purslane extract (400 μg/mL). Western blotting results showing ER stress markers including (**C**) spliced XBP1 (sXBP1; 56 kDa), (**D**) cleaved ATF6 (50 kDa), (**E**) phospho (p)-eIF2α at Ser52 (38 kDa) and (**F**) CHOP (27 kDa) normalized to their total proteins or GAPDH. Data are expressed as the mean ± SEM (*n* = 4). # *p* < 0.05 HG vs. NG; ∗ *p* < 0.05 HG + Purslane vs. HG.

Treatment with high glucose (30 mM, 48 h) downregulated the phosphorylation of eNOS at Ser1177 and phosphorylation of AMPKα at Thr172 (Figure 5A,B), while it upregulated the phosphorylation of eIF2α at Ser52 as well as the expressions of sXBP1, cleaved ATF6 and CHOP (Figure 5C–F). All these changes in protein expressions were reversed by co-treatment of purslane extract (400 μg/mL) (Figure 5 A–F).

### 3.5. Purslane Extract Increases NO Bioavailability via Enhancing AMPK/eNOS Pathway and Reducing ER Stress in RAECs

**Figure 6:**
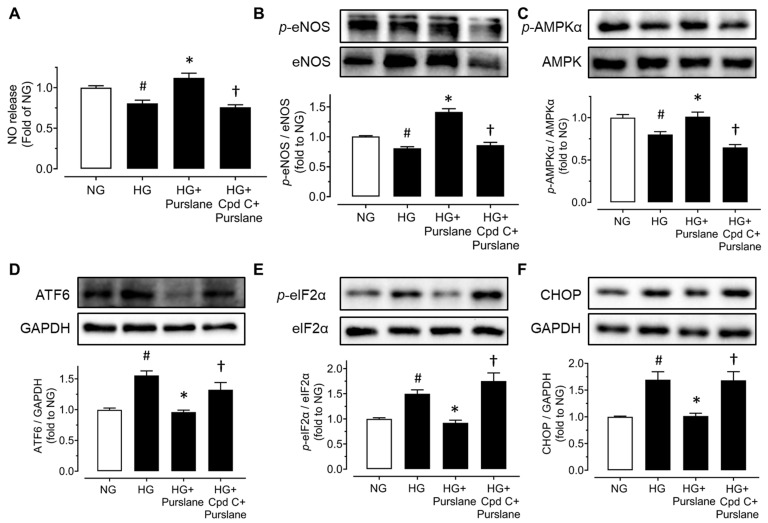
Purslane extract activates AMPKα/eNOS pathway and attenuates ER stress in high glucose-stimulated rat aortic endothelial cells (RAECs). (**A**) NO release from RAECs treated with normal glucose (11 mM present in RPMI-1640 medium), high glucose (44 mM), purslane extract (400 μg/mL), and Compound C (5 μM) for 48 h. Representative blots and summarized data showing (**B**) p-eNOS at Ser1177 (140 kDa), (**C**) p-AMPKα at Thr172 (62 kDa), (**D**) cleaved ATF6 (50 kDa), (**E**) p-eIF2α at Ser52 (38 kDa), and (**F**) CHOP (27 kDa) in the ratios to their total proteins or GAPDH in cultured RAECs. Data are presented as mean ± SEM (*n* = 4). # *p* < 0.05 HG vs. NG; ∗ *p* < 0.05 HG + Purslane vs. HG; † *p* < 0.05 HG + CpdC + Purslane vs. HG + Purslane.

RAECs were treated with a risk factor, high glucose (44 mM, 48 h) or tunicamycin (Tuni, 2 μg/mL, 24 h), and purslane extract (400 μg/mL). Compound C (Cpd C, 5 μM) was also used to reveal the role of AMPK in the vaso-protective effects of purslane extract. High glucose stimulation to the aortic endothelial cells obviously decreased nitrite production, which indicated that the NO bioavailability in RAECs was diminished. Co-treatment with purslane increased NO release whereas Compound C inhibited the increase (Figure 6A). Exposure to high glucose suppressed the phosphorylation of eNOS at Ser1177 and phosphorylation of AMPKα at Thr172 but elevated the expressions of phosphorylated eIF2α (at Ser52), cleaved ATF6 and CHOP. Apparently, co-treatment of purslane extract (400 μg/mL) reversed all these alterations, indicating its vaso-protective effects via activating the AMPK/eNOS pathway and reducing ER stress. Meanwhile, such effects of purslane extract were inhibited by Compound C (Figure 6B–F). 

**Figure 7:**
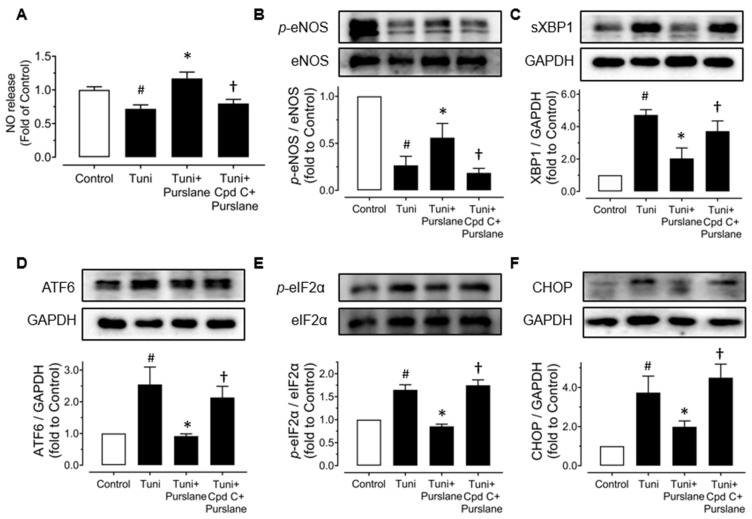
Purslane extract increases eNOS phosphorylation and attenuates ER stress in RAECs exposed to tunicamycin. RAECs were cultured with ER stress inducer tunicamycin (Tuni, 2 μg/mL), purslane extract (400 μg/mL), and Compound C (5 μM) for 24 h. (**A**) Nitrite concentrations representing NO release of RAECs. (**B**) Phosphorylation of eNOS at Ser1177 (140 kDa) compared to total eNOS. Expressions of ER stress markers: (**C**) spliced XBP1 (56 kDa), (**D**) cleaved ATF6 (50 kDa), (**E**) phosphorylation of eIF2α at Ser52 (38 kDa), and (**F**) CHOP (27 kDa) compared to GAPDH or total protein. Data are mean ± SEM of 4–5 experiments. # *p* < 0.05 vs. Control; ∗ *p* < 0.05 vs. Tuni; † *p* < 0.05 vs. Tuni + Purslane.

Similarly, purslane prevented the decrease of nitrite production in tunicamycin-treated RAECs and Compound C inhibited the beneficial effect of purslane (Figure 7A). Upon tunicamycin stimulation, the phosphorylation of eNOS at Ser1177 was significantly decreased and the ER stress markers including sXBP1, cleaved ATF6, eIF2α phosphorylation at Ser52 and C/EBP homologous protein (CHOP) were increased. Purslane extract treatment reversed these alterations and co-treatment with Compound C abolished the protective effects (Figure 7B–F). 

### 3.6. Purslane Extract Suppresses Oxidative Stress via an AMPK Dependent Mechanism

**Figure 8:**
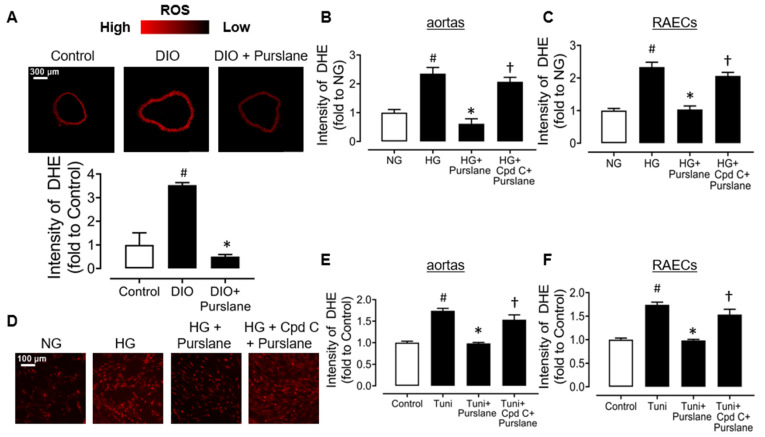
Purslane extract inhibits oxidative stress in aortas and endothelial cells in vivo and in vitro. (**A**) Representative fluorescence images and analyzed data of DHE intensity detected in mouse aortas from lean control and DIO mice with 4-week treatment of purslane extract. (**B**–**D**) DHE intensity in mice aortas and RAECs treated with normal glucose (5.55 mM for aortas, 11 mM for RAECs), high glucose (30 mM for aortas, 44 mM for RAECs), purslane extract (400 μg/mL) and AMPK inhibitor Compound C (0.5 μM), shown as summarized graphs and representative images. (**E**,**F**) Summarized graphs showing the suppressive effects of Purslane extract on tunicamycin (2 μg/mL)-induced oxidative stress in mice aortas and RAECs, which were abolished by Compound C. Data are mean ± SEM of four experiments. # *p* <0.05 vs. Control/NG; ∗ *p* <0.05 vs. DIO/HG/Tuni; † *p* <0.05 vs. HG/Tuni + Purslane extract.

It was observed that the elevated ROS levels in the aortas of DIO mice were inhibited by 4-week purslane extract oral administration (Figure 8A). It was further proved that purslane extract co-incubation (400 μg/mL) inhibited high glucose (30 mM, 48 h)-stimulated ROS elevation in mouse aortas ex vivo, as well as in high glucose (44 mM, 48 h)-treated RAECs. It is worth noting that the antioxidative activity of purslane extract on high glucose-stimulated mouse aortas and RAECs was reversed by Compound C, indicating that the antioxidative effect of purslane extract depends on its activation on AMPK (Figure 8B–D). Moreover, Tuni (2 μg/mL, 24 h)-induced ROS productions in mouse aortas and RAECs were suppressed by co-treatment of purslane extract (400 μg/mL), and such protection was reversed by Compound C (Figure 8E,F).

## 4. Discussion

In our present study, the protective activities of purslane extract in vascular function and metabolic disorders have been illustrated in vivo and in vitro. The major results of the study include (i) four-week oral administration of purslane extract relieved high blood pressure, glucose intolerance, and dyslipidemia in DIO mice; (ii) 4-week purslane treatment reversed the attenuated endothelium-dependent relaxations in aortas, accomplished by upregulation of AMPK/eNOS phosphorylation and inhibition of ER stress; (iii) the endothelial dysfunction of aortas was mitigated by purslane extract under both hyperglycemic and Tuni-induced ER stress conditions ex vivo; and (iv) the increased ROS generation in aortas of DIO mice and in high glucose/Tuni-stimulated aortas and RAECs was restored by purslane extract. 

Earlier work demonstrated that purslane extract possesses hypoglycemic bioactivity in diabetic rats, accomplished with attenuation of oxidative stress and inflammation in plasma and pancreatic cells [22,23]. Importantly, the antidiabetic property of purslane extract treatment is confirmed by a clinical trial in patients suffering from type 2 diabetes [24]. Purslane also attenuates diabetic nephropathy and renal dysfunction [25]; and alleviates vascular inflammation and endothelial function in aortas from diabetic *db*/*db* mice [20]. A recent meta-analysis of randomized controlled trials reveals that purslane is beneficial to diminish FBG levels [26]. Of note, in these previous studies, doses of 100, 200, 250, 300, and 400 mg/kg/day of purslane extract were given whereas the time of treatment was either 4 weeks or 10 weeks. Among them, the low dose of 100 mg/kg/day showed only minor effects. The short-term of 4 weeks and the dose of 200 mg/kg/day for purslane treatment have already shown beneficial effects. Therefore, this dose and time were chosen for the current study. In agreement with the previous studies, our present study showed that a 4-week administration of purslane improved glucose tolerance and insulin sensitivity in obese diabetic mice. Purslane treatment also greatly reduced levels of plasma lipids such as total cholesterol, triglycerides, and LDL cholesterol. This study is probably the earliest to verify that endothelial dysfunction induced by hyperglycemia and obesity could be ameliorated by purslane extract through enhancement of AMPK/eNOS signaling pathways and inhibition on ER stress as well as oxidative stress in vivo, ex vivo, and in vitro. Compound C, a selective AMPK inhibitor, blocked the vaso-protection of purslane extract, showing that endothelial protective activity of purslane works via an AMPK-dependent mechanism. Additionally, according to the results of the Griess assay, purslane extract was effective in increasing NO bioavailability in aortic endothelial cells, which contributes to endothelial function improvement. Hypertension is associated with obesity and diabetes [27]. The beneficial effect of purslane on vascular function was indicated by the lowered blood pressure in obese diabetic mice. The ability of purslane extract to regulate glucose and lipid metabolism might partially conduce to protect vascular function in vivo, and the underlying mechanisms modulating glucose and lipid metabolism are worthy for us to further investigate in future research.

It has been widely known that activation of AMPK is implicated in regulating vascular biology with diverse targets, among which serine 1177 residue of eNOS can be phosphorylated and activated by AMPK directly to promote subsequent NO generation [28]. NO plays an irreplaceable role in vascular homeostasis including modulating vascular tone and protecting endothelial function [29]. Previous studies have reported that endothelial dysfunction in obese mice or rats could be improved through upregulating the AMPK/eNOS pathway [30,31]. Using Compound C in vitro and ex vivo, the elevation of AMPK/eNOS phosphorylation and relevant increase of NO bioavailability by purslane extract were suppressed in our present study. These results illustrated that the AMPK/eNOS/NO signaling pathway is a crucial mechanism involved in the effect of purslane extract to ameliorate endothelial dysfunction. 

Comprehensive evidence has supported the roles of ER stress in vascular function [8,11,32]. ER stress referring to the accumulation of misfolded proteins within the ER due to overwhelmed protein folding capacity, leads to activation of a complicated downstream signaling cascade, including phosphorylation of eIF-2α, splicing of XBP1, and translocation of cleaved ATF6 and thereby induces various responsive gene transcriptions. As happened with ER stress induction, CHOP and NADPH oxidases NOX2 and NOX4 are upregulated to stimulate apoptosis and oxidative stress, respectively, whilst eNOS/its phosphorylation expression levels and the resulting NO bioavailability are diminished. Furthermore, increased ROS levels lead to eNOS uncoupling and reduction of NO production [33]. On the other hand, NO can act as an antioxidant to inhibit ROS generation [34]. ER stress, oxidative stress, and NO bioavailability are interplaying factors, modulating vascular function. AMPK activation, for example, by drugs such as rosiglitazone, inhibits ER stress and oxidative stress to protect endothelial function in diabetes [35]. In the current study, we found that purslane extract restored EDRs in aortas exposed to high glucose/Tuni and from DIO mice under an isobaric condition. This vascular benefit was achieved by AMPK activation, which in turn inhibited ER stress and oxidative stress and enhanced NO generation. Both in vitro and in vivo experiments provided solid evidence for the therapeutic potential of purslane extract for combating against diabetic vasculopathy. 

An increasing number of ingredients have been discovered in *Portulaca oleracea* L. (Purslane), including polysaccharides, flavonoids, alkaloids, sterols, fatty acids, and so on [36,37]. According to our results of UPLC-ESI-QTOF-MS/MS analysis, we confirmed the presence of several flavonoids, consisting of quercitrin, kaempferol, luteolin and their respective glycoside products, luteolin 8-C-glucoside, luteolin-7-O-glucoside, quercetin 3-galactoside and kaempferol-3-O-glucoside in the purslane extract. Existing evidence indicates that dietary flavonoids possess protective bioactivities against the pathology of diabetes. Previous studies have illustrated that a number of flavonoids can effectively activate AMPK and/or inhibit ER stress and oxidative stress, protecting vascular function in diabetes and obesity [21,38,39]. Because of the complexity of the extract itself, future extensive efforts are still needed to explore, which component is responsible for the protective effects on the vascular system, or whether it is a combination of several components or their metabolites. 

There are other limitations in the current study as it did not examine the dose-dependent effect of the purslane extract, which still needs to be investigated with at least three active doses in the future. The present study implies the therapeutic potential of purslane extract to combat diabetic vasculopathy; nevertheless, human studies are obviously necessary to confirm the beneficial effects of purslane extract as rodents are different from humans and the in vitro and ex vivo results on isolated cells or organs cannot reflect the active principles of the plant or its metabolites.

## 5. Conclusions

In conclusion, treatment with purslane extract ameliorates endothelial dysfunction in obesity and diabetes mellitus through suppression of ER stress and oxidative stress by activating the AMPK/eNOS signaling pathway. The present findings suggest the therapeutic potential of purslane extract for pharmaceutical use in treating vascular and metabolic dysfunction.

## Figures and Tables

**Table 1:** Identification information of eight polyphenols in the purslane extract by ESI-QTOF-MS/MS. Retention time, observed mass, theoretical mass, predicted chemical formula, observed MS/MS fragment ions, and the identification results and basis of the eight polyphenols were listed.

No.	Retention Time, min	Observed Mass, *m*/*z*	Theoretical Mass, *m*/*z*	Predicted Chemical Formula	Error, ppm	Observed MS/MS Fragment Ions	Method of Identification	Chemical Compound Name/CAS No.
1	4.78	353.0877	353.0873	C_16_H_18_O_9_	1.1	135.0446, 191.0556, 173.0450, 179.0345	Standard comparison	Cryptochlorogenic acid 905-99-7
2	6.25	447.0933	447.0927	C_21_H_20_O_11_	1.3	357.0615, 327.0507, 297.0341	Standard comparison	Luteolin 8-C-glucoside 28608-75-5
3	7.02	463.0890	463.0877	C_21_H_20_O_12_	2.8	300.0281	Standard comparison	Quercetin 3-galactoside 482-36-0
4	7.71	447.0932	447.0927	C_21_H_20_O_11_	1.1	284.0331, 255.0297, 227.0344	PubChem database	Kaempferol-3-O-glucoside 480-10-4
5	7.91	447.0930	447.0927	C_21_H_20_O_11_	0.6	285.0399, 284.0324, 151.0033, 257.0457	PubChem database	Luteolin-7-O-glucoside 5373-11-5
6	9.43	285.0391	285.0399	C_15_H_10_O_6_	−2.8	285.0401, 133.0295, 151.0116, 175.0441, 199.0446	Standard comparison	Luteolin 491-70-3
7	9.43	301.0356	301.0348	C_15_H_10_O_7_	2.7	151.0084, 107.0188, 121.0332, 179.0057	Standard comparison	Quercetin 117-39-5
8	10.69	285.0404	285.0399	C_15_H_10_O_6_	1.8	285.0432, 239.0361, 185.0600	Standard comparison	Kaempferol 520-18-3

**Table 2:** Lipid profile of male C57BL/6 mice on normal (control) or high-fat diet (DIO) with or without 4-week treatment of purslane extract. Data are means ± SEM of 5 mice from each group. # *p* < 0.05 vs. Control; * *p* < 0.05 vs. DIO.

Plasma Levels of	Control	DIO	DIO + Purslane
Total cholesterol (mg/dL)	74.33 ± 8.16	138.4 ± 2.65 #	123.4 ± 2.56 *
Triglycerides (mg/dL)	101.7 ± 4.43	223.7 ± 7.05 #	188.9 ± 9.95
HDL (mg/dL)	129.93 ± 8.89	97.83 ± 20.11	141.92 ± 11.60
LDL (mg/dL)	11.21 ± 1.55	93.19 ± 5.41 #	26.68 ± 3.09 *

## Data Availability

The data presented in this study are available on request from the corresponding author.
